# Salubrinal attenuated retinal neovascularization by inhibiting CHOP-HIF1α-VEGF pathways

**DOI:** 10.18632/oncotarget.20431

**Published:** 2017-08-24

**Authors:** Yaguang Hu, Xi Lu, Yue Xu, Lin Lu, Shanshan Yu, Qiaochu Cheng, Boyu Yang, Ching-Kit Tsui, Dan Ye, Jingjing Huang, Xiaoling Liang

**Affiliations:** ^1^ State Key Laboratory of Ophthalmology, Zhongshan Ophthalmic Center, Sun Yat-sen University, Guangzhou 510000, Guangdong Province, China; ^2^ Department of Ophthalmology, The First Affiliated Hospital of Xi'an Jiaotong University, Xi'an 710061, Shaanxi Province, China

**Keywords:** salubrinal, RNV, CHOP, HIF1α, VEGF

## Abstract

Retinal neovascularization (RNV) related disease is the leading cause of irreversible blindness in the world. The aim of this study is to identify whether salubrinal could attenuate RNV by inhibiting CCAAT/enhancer-binding protein (C/EBP) homologous protein (CHOP)- hypoxia inducible factors 1α (HIF1α) -vascular endothelial growth factor (VEGF) pathways in both mouse retinal microvascular endothelial cells (mRMECs) and oxygen-induced retinopathy (OIR) mouse model. After being treated with salubrinal (20μmol/L) or CHOP-siRNA, mRMECs were exposed to a hypoxia environment. OIR mice were intraperitoneally injected with salubrinal (0.5 mg/kg/day) from P12 to P17. With salubrinal or CHOP-siRNA treatment, the elevated CHOP protein and mRNA levels in hypoxia-induced mRMECs were significantly decreased. HIF1α-VEGF pathways were activated under hypoxia condition, then HIF1α protein was degraded and VEGF secretion was down-regulated after salubrinal or CHOP-siRNA treatment. In OIR mice, the areas of RNV were markedly decreased with salubrinal treatment. Moreover, elevated expressions of CHOP, HIF1α and VEGF in retinas of OIR mice were all reduced after salubrinal treatment. It suggested that salubrinal attenuated RNV in mRMECs and OIR mice by inhibiting CHOP-HIF1α-VEGF pathways and could be a potential therapeutic target for hypoxia-induced retinal microangiopathy.

## INTRODUCTION

Retinal neovascularization (RNV) related diseases, such as diabetic retinopathy (DR) and retinopathy of prematurity (ROP), are the most serious blindness-cause diseases in the world [1, 2]. Anti-vascular endothelial growth factor (VEGF) drugs injection is the most effective therapy for RNV at present. However, infection risk and economic burdens restricted the clinical applications of anti-VEGF injection [3]. Therefore, finding an effective alternative therapy is imperative.

When experienced inadequate oxygen supply, intracellular factors are activated and lead to the formation of new blood vessels [4, 5]. As a compensatory mechanism, the hypoxia-inducible factor (HIF) 1 response was activated to avoid neovascularization. The HIF1 transcription factors are comprised of two subunit, α and β. During normoxic conditions, HIF1α subunit is rapidly degraded after being hydroxylated by prolyl hydroxylase (PHD) 2. However, when suffering from hypoxia, the HIF1α subunit is stabilized, allowing it to dimerize with HIF1β, to form an active transcription factor. VEGF is a well-known target of HIF1α and is one of the most potent pro-angiogenic factors required for neovascularization. Thus, inhibiting the activation of HIF1α-VEGF pathways is an ideal target to attenuate neovascularization.

CCAAT/enhancer-binding protein (C/EBP) homologous protein (CHOP), a pro-apoptotic protein, is intensely activated when pro-survival exertion does not overcome the sustained endoplasmic reticulum (ER) stress. Recently, evidences indicated that the ER stress played a crucial role in vascular complications [6–8], including neovascularization [9]. Pereira and colleagues [10] found that ER stress potentiated HIF-1 activity to transactivate VEGF expression in the human neuroblastoma cell lines NB1691 and SK-N-AS. Furthermore, several studies have demonstrated that ER stress is a potent inducer of VEGF expression [11–14]. These results indicated that ER stress was an upstream regulator of HIF1α-VEGF pathways. Nevertheless, the relationship between CHOP and HIF1α-VEGF pathways is still unknown.

**Figure 1:**
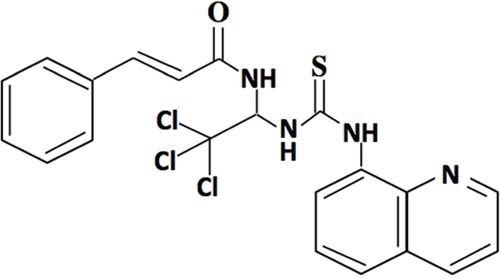
The molecular structure of salubrinal

Salubrinal, a selective dephosphorylation inhibitor of eIF2α, can effectively down-regulate CHOP expression by inhibiting protein kinase RNA-like ER kinase (PERK) pathway [15, 16]. The molecular structural of salubrinal was shown as Figure 1. It had been widely used in protecting ER stress-induced cells apoptosis [17, 18]. And in the neuronal damage caused by chemical hypoxia, salubrinal protected neurons by reducing CHOP levels [19]. Moreover, some studies had reported the protective effect of salubrinal in hypoxic and ischemic diseases, such as global cerebral ischemia [20] and pulmonary hypertension [21, 22]. However, whether salubrinal can regulate HIF1α-VEGF pathways and attenuate neovascularization are still unknown yet.

Herein, for the first time, we demonstrated that salubrinal attenuated retinal neovascularization in hypoxia-induced mouse retinal microvascular endothelial cells (mRMECs) and in retinas of oxygen-induced retinopathy (OIR) mice. Moreover, we demonstrated that the anti-neovascularization effect of salubrinal was with CHOP-HIF1α-VEGF pathways involvement. These results might gain new insight into the clinical treatments of RNV.

## RESULTS

### Salubrinal down-regulates CHOP expression in hypoxia-induced mRMECs

**Figure 2:**
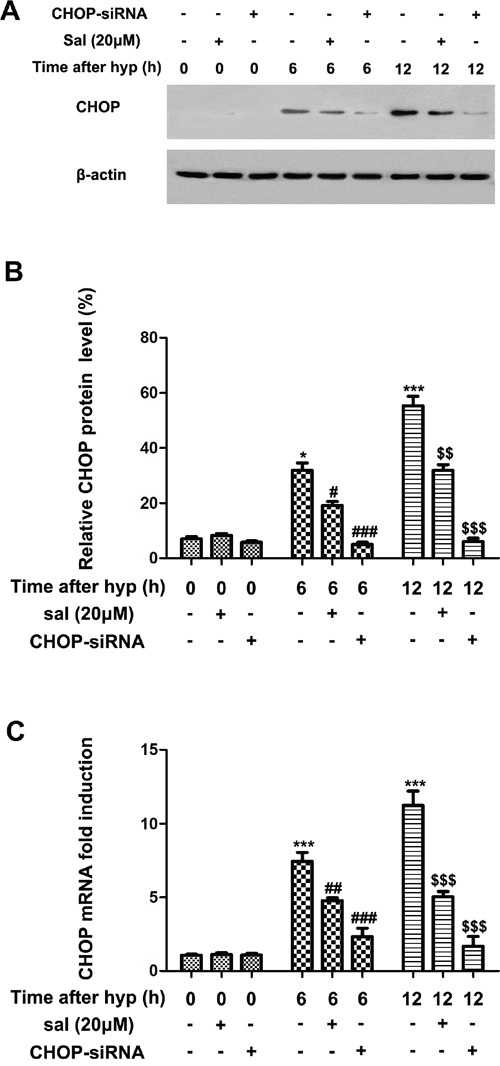
The effect of salubrinal on the activation of CHOP-dependent pathway in the hypoxia-induced mRMECs mRMECs were treated with salubrinal (20μmol/L) or CHOP-siRNA before cultured in hypoxia condition. CHOP protein expression and mRNA level were measured by western blot and RT-PCR, respectively. After hypoxia for 6h and 12h, CHOP protein and mRNA level increasd gradully. With the salubrinal or CHOP-siRNA treatment, the CHOP protein and mRNA level were significantly decreased than mRMECs simply suffering from hypoxia. The results are representative of three independent experiments and the data are expressed as the mean ± standard deviation (*P<0.05 and ***P<0.001, vs. control group; #P<0.05, ##P<0.01 and ###P<0.001 vs. hypoxia group; $$P<0.01 and $$$P<0.001 vs. hypoxia group). mRMEC, mouse retinal micro-vascular endothelial cells; CHOP, C/EBP homologus protein; CHOP-siRNA, CHOP small interfering RNA.

Recent evidences indicated that hypoxia elevated CHOP expression in endothelial cells [23, 24]. Therefore, we investigated whether salubrinal could inhibit the increased CHOP expression in hypoxia-induced mRMECs. As shown in Figure 2A & 2B, hypoxia-induced mRMECs triggered elevated protein level of CHOP at 6h and 12h. Consistent with the change of protein level, the CHOP mRNA level was also promoted by hypoxia (Figure 2C). Moreover, with the treatment of salubrinal (20μmol/L) or CHOP-siRNA, CHOP protein expression was decreased comparing with the hypoxia group at the same time point (Figure 2A & 2B). Besides, the changes of CHOP mRNA were in accordance with the protein changes after the treatment of salubrinal (20μmol/L) or CHOP-siRNA treatment (Figure 2C). All together, these results suggested that in mRMECs, salubrinal might down-regulate the hypoxia-induced CHOP expression.

### Salubrinal promotes degradation of HIF1α in hypoxia-induce mRMECs

**Figure 3:**
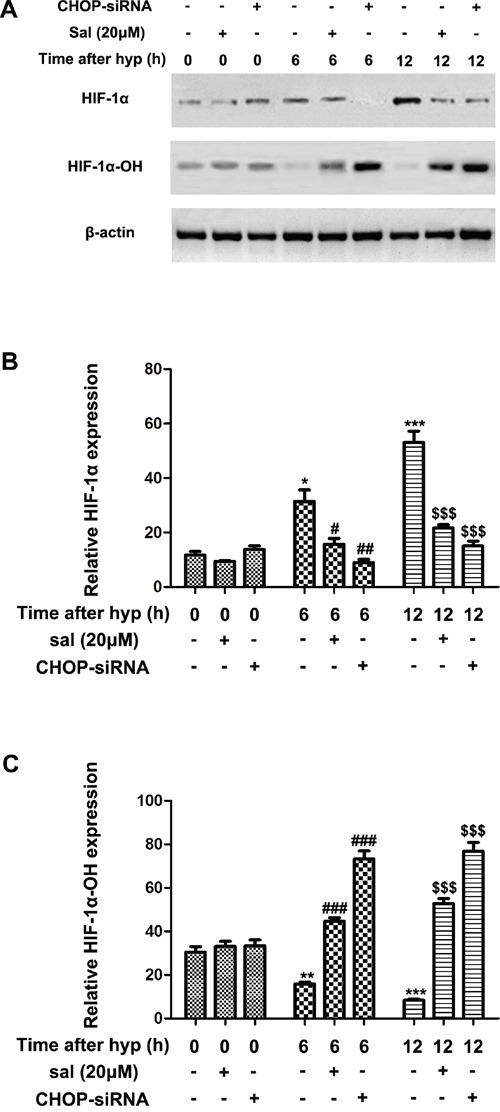
The effect of salubrinal on the hydroxylation of HIF1α in the hypoxia-induced mRMECs mRMECs were treated with salubrinal (20μmol/L) or CHOP-siRNA before cultured in hypoxia condition. Protein expression of HIF1α and HIF1α-OH were measured by western blot. After hypoxia for 6h and 12h, HIF1α protein expression increasd gradully and HIF1α-OH pretein expression reduced gradully at the same time points. Then, with the salubrinal or CHOP-siRNA treatment, HIF1α protein expression was significantly decreased and the HIF1α-OH protein expression was significantly elevated than mRMECs simply suffering from hypoxia. The results are representative of three independent experiments and the data are expressed as the mean ± standard deviation (*P<0.05, **P<0.01 and ***P<0.001, vs. control group; #P<0.05, ##P<0.01 and ###P<0.001 vs. hypoxia group; $$$P<0.001 vs. hypoxia group). mRMEC, mouse retinal micro-vascular endothelial cells; CHOP-siRNA, C/EBP homologus protein small interfering RNA; HIF, hypoxia-inducible factor.

Quantity researches revealed that HIF1α-VEGF pathways were primarily participated in hypoxia stress [25–27]. To further investigate whether salubrinal affected the elevated HIF1α expression in hypoxia-induced mRMECs, we treated the mRMECs with salubrinal (20μmol/L) or CHOP-siRNA. As expected, HIF1α protein expression significantly increased at 6h and 12h after hypoxia (Figure 3A & 3B). And HIF1α-OH protein expression decreased as the stability of HIF1α increased under hypoxia condition (Figure 3A & 3C). Salubrinal or CHOP-siRNA treatment significantly reduced HIF1α protein expression and elevated HIF1α-OH protein expression (Figure 3). These results suggested that HIF1α protein was unstable and easily to be degraded after salubrinal down-regulating the CHOP expression in hypoxia-induced mRMECs.

### Salubrinal inhibits VEGF secretion in hypoxia-induced mRMECs

**Figure 4:**
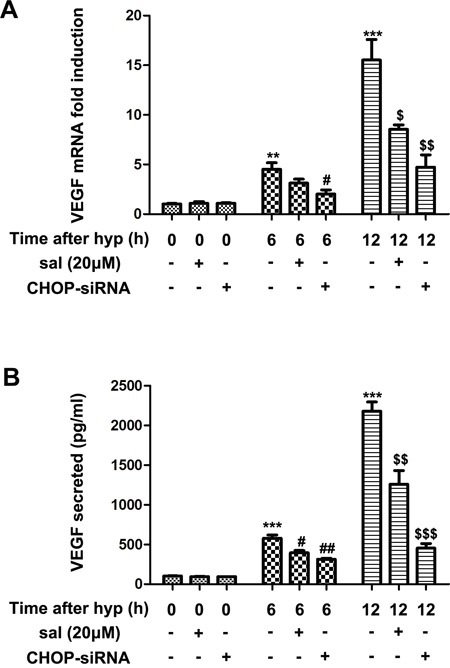
The effect of salubrinal on the expression of VEGF in the hypoxia-induced mRMECs Following salubrinal (20μmol/L) treatment or CHOP-siRNA transfection, mRMECs were cultured in hypoxia condition for 6h and 12h. VEGF mRNA experssion level was measured by RT-PCR **(A)** and VEGF secretion level was measured by ELISA **(B)**. With the salubrinal and CHOP-siRNA pretreatment, the elevated protein expression and mRNA level were both decreased. The results are representative of three independent experiments and the data are expressed as the mean ± standard deviation (**P<0.01 and ***P<0.001, vs. control group; #P<0.05, ##P<0.01 vs. hypoxia group; $P<0.05, $$P<0.01 and $$$P<0.001 vs. hypoxia group). VEGF, vascular endothelia growth factor; mRMEC, mouse retinal micro-vascular endothelial cells; CHOP-siRNA, C/EBP homologus protein small interfering RNA; RT-PCR, reverse transcription-polymerase chain reaction; ELISA, enzyme-linked immunosorbent assay.

Mounting evidence revealed that VEGF was a well-known HIF1α target and it was markedly upregulated under hypoxic conditions [28–30]. Thus, to further explore whether salubrinal could inhibit VEGF expression, we investigated the transcription and secretion of VEGF after treating the hypoxia-induced mRMECs with salubrinal or CHOP-siRNA. VEGF mRNA was quantified for VEGF transcription by RT-PCR analysis, and culture supernatants were examined for VEGF secretion by ELISA. We found that VEGF secretion and mRNA level were gradually increased at 6h and 12h after hypoxia (Figure 4). Treating the mRMECs with salubrinal (20μmol/L) or CHOP-siRNA significantly down-regulated VEGF mRNA level (Figure 4A) and reduced VEGF secretion (Figure 4B). All together, these results further suggested that salubrinal might inhibit CHOP-HIF1α-VEGF pathways in hypoxia-induced mRMECs.

### Salubrinal inhibits RNV in OIR mice

**Figure 5:**
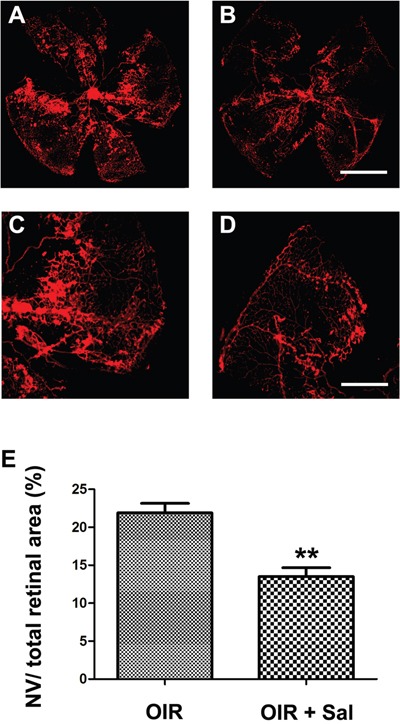
The effect of salubrinal on the areas of RNV in OIR mice evaluated by whole-mount immunostaining Mice retinas from OIR group **(A, C)** and OIR+ salubrinal (0.5 mg/kg/day) group **(B, D)** were harvested at P17 and subjected to whole-mount immunostaining. The areas of RNV were markedly attenuated with the treatment of salubrinal **(E)**. Scale bar = 500μm (**P<0.01 vs. OIR group). RNV, retinal neovascularizaiton; OIR, oxygen-induced retinopahty.

As the results shown, salubrinal inhibited CHOP-HIF1α-VEGF pathways in hypoxia-induced mRMECs. So whether salubrinal affected the retinal neovascularization in OIR mice was evaluated with immunostaining on whole-mount retinas. The retinas of OIR mice showed obvious neovasculars and a multiple of neovasculars tufts covering at the leading edge of retinal vessels (Figure 5A & 5C); however, the areas of neovasculars and neovasculars tufts were significantly reduced with the treatment of salubrinal (Figure 5B & 5D).

**Figure 6:**
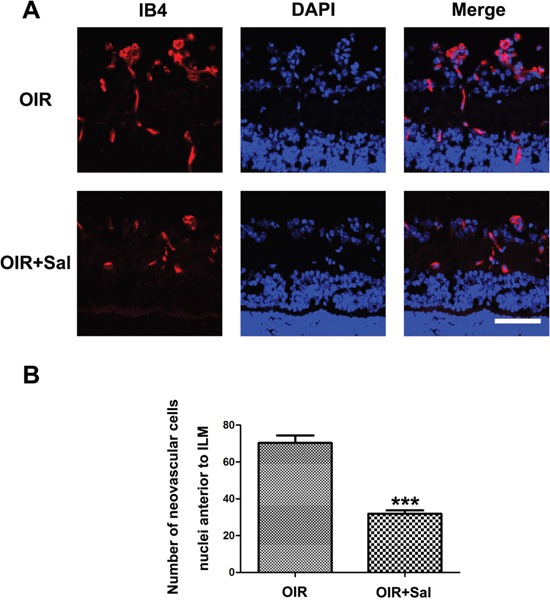
The effect of salubrinal on the areas of RNV in OIR mice evaluated by frozen sections immunostaining Mice retinas from OIR group and OIR+ salubrinal (0.5 mg/kg/day) group were harvested at P17 and subjected to frozen sections immunostaining. RNV **(A)** and the numebers of RNV cell nuclei anterior to ILM **(B)** were significantly drcreased with the salubrinal treatment. Scale bar = 100μm. (***P<0.01 vs. OIR group). RNV, retinal neovascularizaiton; OIR, oxygen-induced retinopahty; ILM, internal limiting membrane.

To further identify the anti-neovascularization effect of salubrinal, retinal frozen sections were performed. As shown in Figure 6A, the neovasculars in OIR mice were markedly attenuated with the salubrinal treatment. Furthermore, salubrinal significantly reduced the numbers of neovasculars cell nuclei anterior to internal limiting membrane (ILM) in OIR mice (Figure 6B). These results even more indicated that salubrinal could dramatically inhibit the RNV in OIR mice.

### Salubrinal down-regulates CHOP expression in retinas of OIR mice

**Figure 7:**
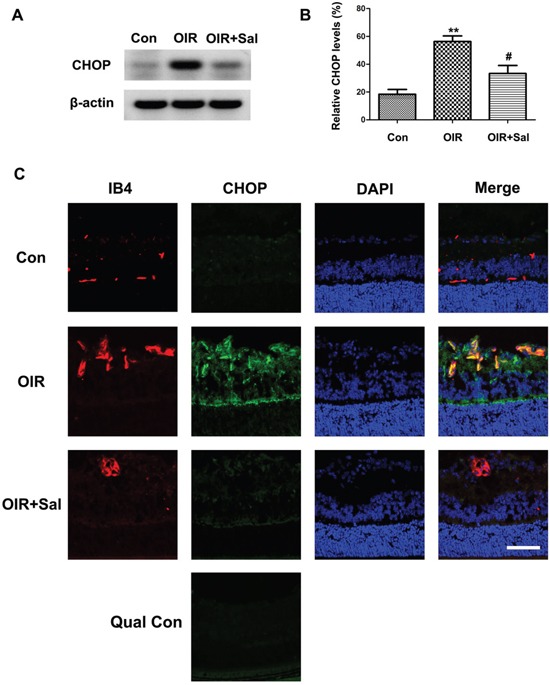
The effect of salubrinal on the activation of CHOP-dependent pathway in OIR mice Mice retinas from OIR group and OIR+ salubrinal (0.5 mg/kg/day) group were harvested at P17, and then the retians were subjected to western blot and frozen sections immunostaining. The werstern blot results showed that, with salubrinal treatment, the elevated CHOP protein expression in OIR group was significantly decreased **(A** and **B).** The immunostaining results showed that the neovascularization and elevated CHOP protein expression in OIR group were significantly decreased **(C).** The immunostaining images with omission of the primary antibody was provided as quality control. Scale bar = 100μm. The results are representative of three independent experiments and the data are expressed as the mean ± standard deviation (**P<0.01, vs. control group; #P<0.05, vs. OIR group). OIR, oxygen-induced retinopahty; CHOP, C/EBP homologus protein.

Our study showed that in hypoxia-induced mRMECs, salubrinal could down-regulated CHOP expression. Thus, we investigated whether salubrinal inhibited RNV by the CHOP-dependent pathways. As shown in Figure 7A & 7B, CHOP proteins were strongly expressed in retinas of OIR mice. Furthermore, the immunostaining results also showed that expression of CHOP proteins were increased and most of them were located in IB4-positive vascular endothelial cells (Figure 7C). However, CHOP expression was significantly decreased with salubrinal treatment. These results suggested that salubrinal inhibited RNV through down-regulating CHOP expression in retinas of OIR mice.

### Salubrinal down-regulates the activation of HIF1α-VEGF pathway in retinas of OIR mice

**Figure 8:**
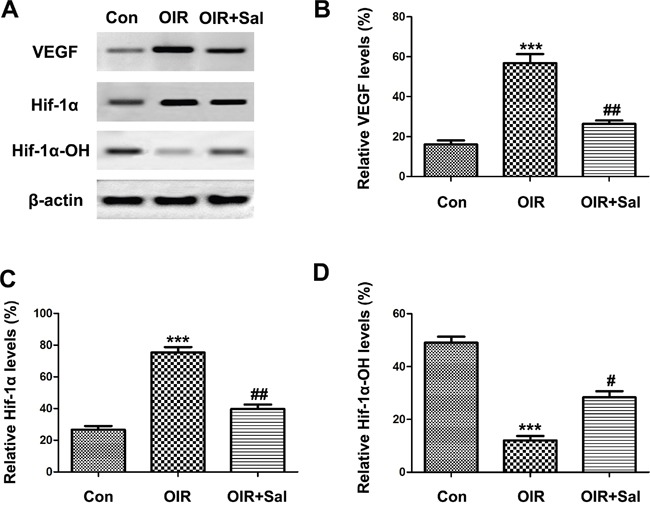
The effect of salubrinal on the activation of HIF1α-VEGF pathway in OIR mice Mice retinas from each group were harvested at P17 and subjected to western blot. The elevated HIF1α and VEGF protein expressions in OIR group were decreased significantly with the treatment of salubrinal. And HIF1α-OH protein expression was increased as the stability of HIF1α inhibited by salubrinal. The results are representative of three independent experiments and the data are expressed as the mean ± standard deviation (***P<0.001, vs. control group; #P<0.05, ##P<0.01 vs. OIR group). OIR, oxygen-induced retinopahty; HIF, hypoxia-inducible factor; VEGF, vascular endothelia growth factor.

HIF1α-VEGF pathway had been well studied in RNV [31–33]. Thus, we investigated whether salubrinal could down-regulated HIF1α-VEGF pathway in retinas of OIR mice. As the western blot results shown, the expressions of HIF1α and VEGF were up-regulated in retinas of OIR mice, which were attenuated in salubrinal-treated OIR mice (Figure 8A & 8B & 8C). As the stability of HIF1α decreased, the protein expression of HIF1α-OH elevated with the salubrinal treatment (Figure 8A & 8D). All together, these results suggested that CHOP-HIF1α-VEGF pathway was involved in salubrinal-mediated anti-neovascularazation effects in OIR mice.

## DISCUSSION

RNV is a major cause of blindness in the world. Responding to hypoxia, RNV grows within retina and subsequently breakthrough the retina surface to cause vitreous hemorrhage and tractional retinal detachment [34]. Anti-VEGF drugs are the most effective anti-neovascularization therapy at present. However, they are restricted in some degree. Thus, going deeper into the pathogenesis of RNV and finding out an innovative therapy are extremely urgent at once. Salubrinal, a pharmacological inhibitor of eIF2α dephosphorylation, is known to possess a variety of biological effects, including anti-apoptosis [17, 18], anti-arrhythmia [35], anti-thermal injury [36] and anti-oxidant [37]. Furthermore, some studies had reported the protective effect of salubrinal in hypoxic and ischemic diseases [21, 22]. However, whether salubrinal can inhibit hypoxia-induced neovascularization in retina is unknown yet.

Hypoxia-cultured mRMECs and OIR mice model had been established in our previous studies [38–40]. Both of them are useful and intensively investigated models for studying the molecular mechanisms of RNV. OIR mice model exhibits two stages. During the first hyperoxic phase, vascular development arrests, existing blood vessels degenerate and form the vaso-obliterated zones in central retina [41, 42]. During the secondary hypoxic phase, the avascular retina becomes ischemic and triggers a compensatory release of pro-angiogenic factors, such as VEGF, which induces RNV [41, 42]. In our study, we found that after the treatment of salubrinal, areas of peripheral pathological RNV significantly attenuated. And frozen sections were performed to identify the anti-neovascularization effect of salubrinal. Furthermore, we demonstrated that the anti-neovascularization role of salubrinal in retinas of OIR mice was accomplished through inhibiting the activation of CHOP-HIF1α-VEGF pathways. In addition, we found that salubrinal or CHOP-siRNA promoted degradation of HIF1α and down-regulated the secretion of VEGF by inhibiting CHOP-dependent pathways in hypoxia-induced mRMECs.

As compensatory response under hypoxia, HIF1α-VEGF was activated to restore and enhance vascularization. Such biological activity can happen in physiological conditions, like wound healing [43, 44]. HIF1α is a master regulator of oxygen homeostasis [45]. Usually, HIF1α is present at undetectable levels under normoxic conditions because of rapid hydroxylation, however this process is inhibited under hypoxic conditions [46, 47]. Tina Friis et al.[48] demonstrated that salubrinal has an influence on angiogenic process, consisting of inhibiting VEGF signaling and endothelia cells proliferation and migration. In our study, we found that both *in vivo* and *in vitro*, HIF1α protein was promoted to degrade and the expression of VEGF was significantly down-regulated with the treatment of salubrinal. These results suggested that salubrinal prevented hypoxia-induced RNV by blocking HIF1α-VEGF pathways.

Endoplasmic reticulum (ER) is a principal organelle almost existed in all eukaryotic cells. Hypoxia disturbs ER homeostasis, resulting in the accumulation of unfolded or misfolded proteins and inducing ER stress in endothelial cells [23]. ER stress has three main signaling cascades, PERK-eIF2a, IRE1a, and ATF6. Pereira and her colleagues [10] found that ER stress potentiated HIF1α activity to transactivate VEGF expression in tumor cells. Recently, Jiang X et al. [49] showed that in ARPE-19 cells, ER stress was induced by thapsigargin treatment. Then, CHOP and VEGF expressions were both down-regulated after inhibiting PERK-eIF2a pathways by a highly selective inhibitor. Furthermore, a clear interaction between CHOP, a proapoptotic protein works at the converging point of PERK-eIF2a pathways, and VEGF is also revealed in multiple diseases [11, 50–54]. In our study, we use salubrinal or CHOP-siRNA to inhibit the CHOP-dependent pathways in hypoxia-induced mRMECs and OIR mice to explore the relationship between CHOP and HIF1α-VEGF pathways. *in vivo*, we found that with the treatment of salubrinal, the HIF1α and VEGF expressions were significantly down-regulated after CHOP expression was silenced. Consistent with the results *in vivo*, after the salubrinal treatment, HIF1α and VEGF expressions in retinas of OIR mice were markedly reduced after the CHOP expression decreased. There results suggested that CHOP could regulate the HIF1α-VEGF pathways in hypoxia-induced mRMECs and in retinas of OIR mice.

In conclusion, for the first time, our study provided evidences that salubrinal attenuated hypoxia-induced retinal neovascularization via inhibiting CHOP-HIF1α-VEGF pathways. Therefore, salubrinal is a potential candidate to prevent RNV, and further studies are necessary to assess the salubrinal anti-neovascularization effect by gene knockout mouse model.

## MATERIALS AND METHODS

### Reagents

The mRMECs were purchased from Cell Biologics (Chicago, USA). The fetal bovine serum (FBS), endothelial cell medium (ECM) were purchased from Gibco (NY, USA). The bovine serum albumin (BSA), phosphate-buffered saline (PBS) solution, Tris-HCl buffer salt solution (TBS), Tween-20, TritonX-100, dimethylsulfoxide (DMSO) and salubrinal were purchased from Sigma Chemical Co. (St. Louis, MO, USA). The penicillin, streptomycin and Oligofectamine were purchased from Thermo Fisher Scientific (Bremen, Germany). The red-light-absorbing dye labeled Griffonia simplicifolia isolectin B4 (IB4) and TRIzol were purchased from Invitrogen Life Technologies Co. (Carlsbad, CA, USA). The polyvinylidene difluoride filter (PVDF) membrane and chemiluminescence system were purchased from Millipore (Millipore, CA, USA). The skim milk, paraformaldehyde (PFA), DAPI, RIPA, phenylmethanesulfonyl fluoride (PMSF) and BCA protein assay were purchased from Beyotime Institute of Biotechnology (Shanghai, China). Antibodies against CHOP, HIF1α, β-actin, goat anti-rabbit conjugated with horseradish peroxidase (HRP) secondary antibody and Alexa Fluor 488-labeled goat anti-mouse secondary antibody were purchased from Cell Signaling (Beverly, CA, USA). Antibody against HIF1α-OH was purchased from Abnova (Taiwan, China). The vascular endothelial growth factor (VEGF) enzyme-linked immunosorbent assay (ELISA) kit was purchased from R&D system (Minneapolis, MN, USA). The O.C.T. compound was purchased from Sakura Finetek (Torrance, CA, USA). SYBR Green RT-PCR Master mix was purchased from Biotool (Houston, USA). CHOP-small interfering RNA (CHOP-siRNA) was purchased from Ribobio (Guangzhou, China).

### Animals

C57BL/6J mice were obtained from Animal Laboratory of Zhongshan Ophthalmic Center (Guangzhou, China). Care, use and treatment of all mice in this study were in strict agreement with the Statement for the Use of Animals in Ophthalmic and Vision Research from the Association for Research in Vision and Ophthalmology and were approved by the Institutional Animal Care and Use Committee of Zhongshan Ophthalmic Center.

### Mouse model of oxygen-induced retinopathy and treatment

OIR mouse model was established as described in our previous studies [40, 55, 56]. Briefly, newborn C57BL/6J mice at postnatal day 7 (P7) were exposed to hyperoxia (75% O_2_) for 5 days and then returned to normoxia (room air) at P12, whilst control groups were maintained constantly in room air. Then, OIR mice at P12 were divided into two groups: OIR + salubrinal group (intraperitoneal injected with salubrinal, 0.5 mg/kg/day, the dosage was based on previous study [57], from P12 to P17), a vehicle group (intraperitoneal injected with an equal volume of DMSO from P12 to P17).

### mRMECs culture and treatment

mRMECs were cultured in ECM, supplemented with 10% FBS, 100U/ml penicillin, 100U/ml streptomycin, and maintained at 37°C in a humidified atmosphere of 5% CO_2_ and 95% air. The cells used in the present study were between passage 3-5. Once the cells had grown to 70% confluence, they were treated with salubrinal (20μmol/L) for 1h or transfected with siRNA targeting CHOP using Oligofectamine according to the protocol. The sequence of CHOP-siRNA was 5’-GAGCUCUGAUUGACCGAAUGGUGAA-3’, which was adopted according to previous published work [20]. Then, mRMECs were cultured under hypoxia condition (93% N_2_, 5% CO_2_, 1% O_2_) for 6h and 12h. Then the cells were washed twice with PBS for subsequent experiments.

### Western blot

mRMECs and retinas were lysed on ice using RIPA buffer containing a protease inhibitor PMSF (1:100). Protein concentration was quantified by BCA protein assay. Total protein was fractionized by electrophoresis in 10% sodium dodecyl sulfate-polyacrylamide gels and transferred to PVDF membranes. The membranes were blocked in 5% skim milk in TBST for 2h at room temperature and incubated with primary antibody against CHOP (anti-rabbit, 1:500), HIF1α (anti-rabbit, 1:500), HIF1α-OH (anti-rabbit, 1:500) at 4°C overnight. β-actin (anti-rabbit, 1:1000) was used as loading control. The membranes were then washed in TBST and incubated with second antibody goat anti-rabbit conjugated with HRP (1:5000) for 2h at room temperature. The immunoreactive bands were visualized using an enhanced chemiluminescence system.

### Real-time PCR (RT-PCR) analysis

Total RNA was purified from mRMECs with TRIzol, and converted to complementary DNA (cDNA) by using a cDNA first-strand synthesis system (Fermentas, Canada). RT-PCR was performed using the SYBR Green RT-PCR Master mix, according to manufacturer's protocol. The cycle threshold values were normalized against β-actin and the 2-ΔΔCq method was used to calculate target gene expression. The PCR primers were designed based on the NCBI GeneBank database. The primers for mice CHOP, VEGF and β-actin were as shown in Table 1. All reactions were run in triplicate.

**Table 1:** Primers of mice CHOP, VEGF and β-actin for real-time PCR

Primers		Sequence(5’-3’)
CHOP	Forward	5'-CAC CTA TAT CTC ATC CCC AGG AAA CG-3'
	Reverse	5'-TTC CTT GCT CTT CCT CCT CTT CCT CC-3'
VEGF	Forward	5'-GCA CAT AGG AGA GAT GAG CTT CC-3'
	Reverse	5'-CTC CGC TCT GAA CAA GGC T-3'
β-actin	Forward	5'-GGC GGA CTA TGA CTT AGT TT-3'
	Reverse	5'-AAA CAA CAA TGT GCA ATC AA-3'

### ELISA

mRMECs were plated into 96-well plates (1×10^5^ cells/well) and cultured at 37°C in a humidified 5% CO_2_ atmosphere for 24h. After various treatments, culture medium supernatants were collected to determinate the VEGF concentrations by ELISA. The level of VEGF was measured using ELISA kit. Each well was immediately read in a microplate reader (LabSystems; Thermo Fisher Scientific, Inc.) at 450 nm. Measurements were repeated in triplicate.

### Immunofluorescent staining on whole-mount retinas

Mice at P17 were euthanized, and eyes were fixed with freshly prepared 4% PFA for 30min. Retinas were carefully dissected and blocked in PBS containing 5% BSA and 1.5% Triton X-100 at 4°C overnight. Subsequently, retinas were incubated with red-light-absorbing dye labeled (Alexa Fluor 568) Griffonia simplicifolia IB4 (1:50, a marker for vessels) overnight at 4°C. Then, retinas were washed with PBS three times and mounted on microscope slides. Retinas were examined by fluorescence microscopy (AxioCam MRC; Carl Zeiss, Thornwood, NY). Areas of avascular and neovascular were quantified using Image J software (National Institutes of Health, Bethesda, MD).

### Immunofluorescent staining on retinal frozen sections

Eyes were enucleated and fixed overnight in 4% PFA. Subsequently, eyes were equilibrated in 30% sucrose and embedded in OCT. Then they were fast frozen and cut into 6μm-thick sections. The sections were washed with PBS, and permeabilized with 1% Triton X-100 for 15min at room temperature before blocking with 1% BSA for 2h. Then, sections were incubated overnight at 4°C with red-light-absorbing dye labeled (Alexa Fluor 568) Griffonia simplicifolia IB4 (1:50) and mouse monoclonal primary antibodies for CHOP (1:200). After washing in PBS, sections were incubated for 2h at room temperature with Alexa Fluor 488-labeled goat anti-mouse secondary antibody (1:500) and were counterstained with DAPI for 10min. The immunostaining images with omission of the primary antibody was provided as quality control. Retinas were examined by confocal microscopy (Zeiss 510; Carl Zeiss).

### Statistical analysis

All data were expressed as mean ± SEM for three independent experiments. In all cases, *P* value < 0.05 was considered statistically significant difference. Student's t test was used when two groups were compared. One-way ANOVA followed by Tukey multiple comparison was used when different groups were compared. Statistical analyses were performed with GraphPad Prism (v6.0, GraphPad Software Inc.).
